# Perioperative Telemedicine Care in Hernia Surgery: A Systematic Review and Meta-Analysis

**DOI:** 10.7759/cureus.92605

**Published:** 2025-09-18

**Authors:** W. Rodrigo Calmet Rocca, Alexandre Oliveira Carneiro, Sergio Mazzola Poli De Figueiredo

**Affiliations:** 1 Faculty of Medicine Alberto Hurtado, Universidad Peruana Cayetano Heredia, Lima, PER; 2 Faculty of Medicine, Universidade Federal de Uberlândia, Minas Gerais, BRA; 3 Surgery, The University of North Carolina at Chapel Hill, North Carolina, USA

**Keywords:** abdominal wall surgery, complications, hernia surgery, surgical site occurrences, telemedicine

## Abstract

Telemedicine has been increasingly incorporated into medical practice in the last few years. However, it is still not a routine practice in our field. There is limited data on the impact of telemedicine on outcomes in hernia surgery. This systematic review and meta-analysis aims to evaluate the outcomes between telemedical perioperative care and in-person traditional care in hernia surgery.

PubMed, EMBASE, and Cochrane were searched for studies comparing Telemedical interventions and traditional In-Person perioperative care for hernia surgery. Postoperative outcomes were assessed by pooled analysis and meta-analysis. Statistical analysis was performed using Review Manager 5.4 (Nordic Cochrane Center, The Cochrane Collaboration, Copenhagen, Denmark).

One thousand and thirty-four articles were screened, and 39 were thoroughly reviewed. We included three observational retrospective studies, with 2,582 patients receiving telemedicine or in-person care for hernia surgery repair. A total of 2,049 (79%) patients received telemedicine care, and 533 (21%) received traditional care. There was no significant differences in overall complications (14% vs. 13%; odds ratio (OR) 1.11; 95% confidence interval (CI) 0.83-1.48; *P *= 0.49; *I*² = 62%), surgical site occurrences (8% vs. 10%; OR 0.91; 95% CI 0.54-1.56; *P *= 0.14; *I*² = 0.48%), and readmission rates (3% vs. 2.6%; OR 1.41; 95% CI 0.19-10.39; *P *= 0.73; *I*² = 53%) between both groups.

In conclusion, perioperative telemedicine in hernia surgery may be safe compared to traditional in-person care, without significant differences in complications, readmissions, or surgical site occurrences. Our findings support telemedical perioperative care as a feasible alternative that enhances patient access while maintaining optimal surgical outcomes. Further high-quality prospective studies are warranted to better establish the role of telemedicine in hernia surgery.

## Introduction and background

Over the last 50 years, the surgical field has rapidly evolved with new technologies, including the use of telemedicine. Although telemedical care has been implemented in multiple specialties globally, there are still cultural dogmas of surgical consultations that have limited its advance within surgical specialties, particularly abdominal wall surgery. [1]

The word *telemedicine* has been defined as a service where physicians can interact remotely with patients using telecommunication technologies [2-4]. Recent evidence has demonstrated that digital health interventions during the perioperative period do not lead to an increase in complications or readmission rates in patients undergoing abdominal surgery [1,5-11]. These findings suggest that telemedicine can be safely incorporated into perioperative care without compromising patient outcomes. However, despite such strong and favorable conclusions, it would be inappropriate to generalize that all candidates to undergo any kind of abdominal surgery should universally receive perioperative telemedicine care.

In patients undergoing hernia repair, postoperative complications are generally infrequent and tend to follow a relatively benign clinical course. Consequently, the routine need for traditional in-person perioperative care has been increasingly questioned [7]. The present study is a systematic review and meta-analysis that evaluates perioperative telemedicine interventions in elective abdominal wall hernia surgery. The included studies primarily report on ventral and inguinal repairs, although most did not consistently distinguish between primary and recurrent cases or specify hernia subtypes. By focusing on this population, we aim to compare postoperative outcomes between patients receiving perioperative telemedicine and those managed through standard in-person care.

## Review

Methodology

Eligibility Criteria

Inclusion in this meta-analysis was restricted to studies that met all the following eligibility criteria: (1) randomized controlled trials, non-randomized controlled trials, or observational studies (cohort and case-control); (2) adult patients undergoing abdominal wall hernia surgery, without sex restrictions; and (3) comparisons of any digital health intervention (e.g., video calls, phone calls) provided perioperatively versus conventional in-person care. The exclusion criteria were (1) studies without a comparable group; (2) interventions that did not involve patient-clinician communication; (3) reviews, commentaries, letters, editorials, case reports, non-peer-reviewed sources, or studies available only in abstract form; and (4) studies not written in English, Spanish, or Portuguese.

Search Strategy and Data Extraction

We systematically searched PubMed, Embase, and Cochrane Central Register of Controlled Trials from inception to December 2024 with the following search terms: "Hernia surgery", "Telemedicine", "Telehealth", "Follow-up". The search was conducted by two authors (RC and AC). The references from all included studies, previous systematic reviews, and meta-analyses were manually reviewed for additional studies. Two authors (RC and AC) independently extracted baseline characteristics and outcomes data following predefined search criteria and quality assessment. Any discrepancy was resolved by discussion in a consensus meeting among the authors. The prospective meta-analysis protocol was registered in the International Prospective Register of Systematic Reviews (PROSPERO) on December 5, 2024, under the identifier CRD42024622694.

Endpoints and Sensitivity Analyses

The primary outcome was the number of postoperative complications. Secondary outcomes were surgical site occurrences (SSOs), defined as any surgical site complication, including surgical site infection (SSI), cellulitis, necrosis, non-healing wounds, seroma, hematoma, dehiscence, or fistula.

This classification of complications was standardized to account for the variability of the descriptions in the types of SSOs reported across the included studies. We also addressed the hospital readmission rate as a secondary outcome. No predefined temporal endpoint for readmission was established.

Quality Assessment

Data were extracted using a standardized extraction form. This form sought information on publication details, design, population, intervention, and outcomes. Two independent authors completed the risk of bias (RC and AC). Disagreements were resolved through a consensus after discussion of the reasons for the discrepancy. Publication bias was investigated by funnel-plot analysis of point estimates in relation to study weights.

Statistical Analysis

This systematic review and meta-analysis were performed and reported in accordance with the Cochrane Collaboration Handbook for Systematic Review of Interventions and the Preferred Reporting Items for Systematic Reviews and Meta-Analysis (PRISMA) statement guidelines [12].

Odds ratios (OR) with 95% confidence intervals (CIs) were used to compare the digital health interventions for categorical endpoints, which are the only ones we are evaluating in this study (number of complications, SSOs, and readmission rates).

Heterogeneity was assessed using Cochran’s Q test, I² statistics, and visual inspection of forest plots, but the choice of model was determined a priori according to our protocol, not based on observed heterogeneity. A random-effects model was used for all analyses, based on the assumption that true effect sizes may vary across studies. Statistical analysis was conducted using Review Manager 5.4 (Nordic Cochrane Center, The Cochrane Collaboration, Copenhagen, Denmark).

Results

Study Selection and Baseline Characteristics

**Figure 1:**
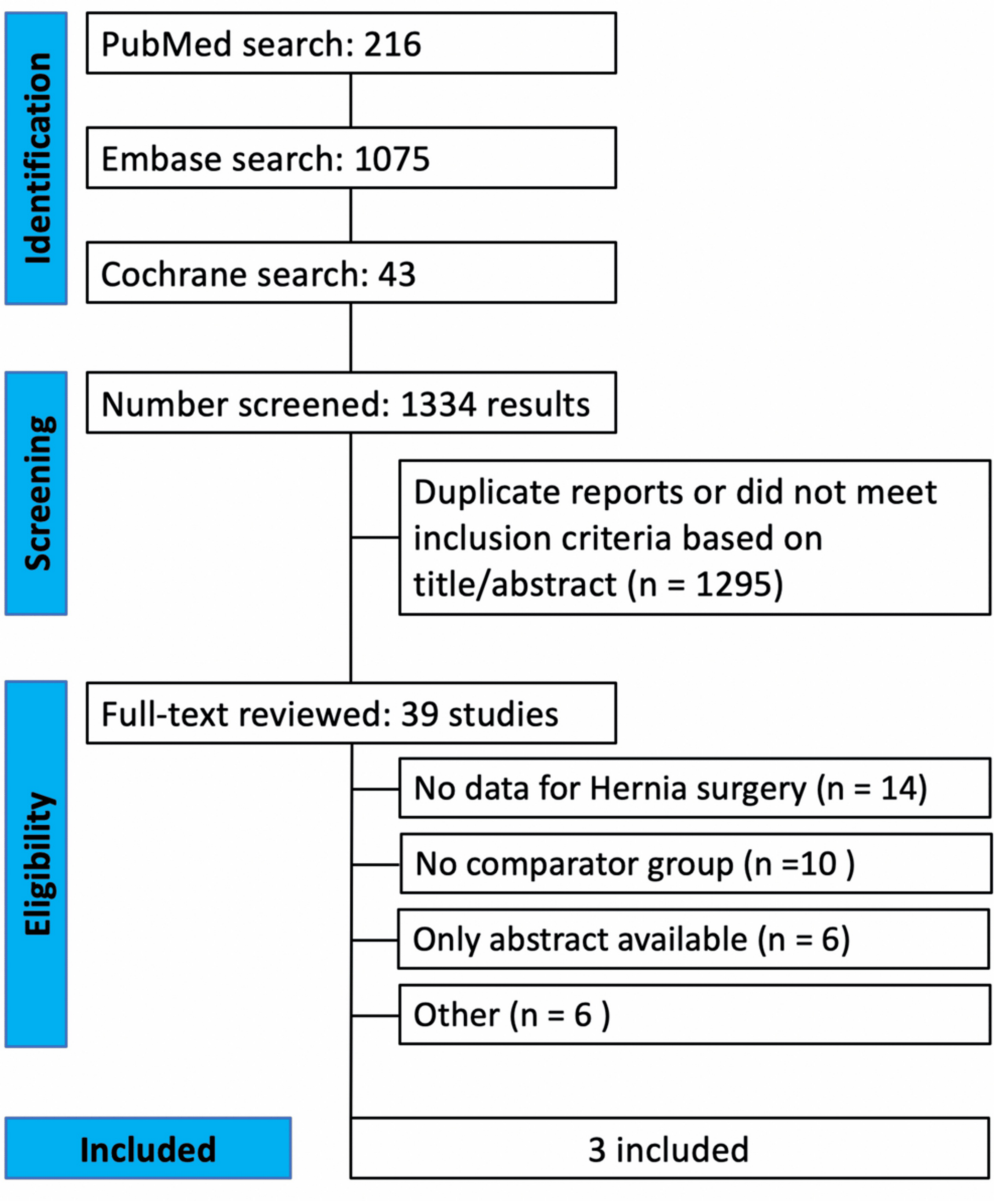
PRISMA flow diagram of study screening and selection. Three articles were included [5-7]. PRISMA, Preferred Reporting Items for Systematic Reviews and Meta-Analysis

The initial search yielded 1,334 results. After removal of duplicate records and excluded studies, 39 were chosen to be fully reviewed based on our inclusion criteria. Of these, a total of three studies were included, since most of those (*n* = 14) did not show data for hernia surgery specifically. These three studies were observational: two case-control and one prospective cohort, including 2,582 patients in total (Figure 1).

The studies were published between 2021 and 2024. All of them (100%) were from the United States. A total of 2,049 (79%) patients received telemedicine interventions during their perioperative care, and 533 (21%) had traditional in-person care. Study characteristics are described in Table 1.

**Table 1:** Studies included, demographics, and operative characteristics of the patients. *Age and BMI are reported as provided in the original studies. Values may be ranges with percentages [6], median with Q1-Q3 and range [7], or median (IQR) [5]. SD was not reported in any study. BMI, body mass index; SD, standard deviation; IQR, interquartile range

Characteristics	Liu et al. (2021) [6] (*N* = 2009)			Abbitt et al. ( 2023) [7] (*N* = 338)		Felix et al. (2024) [5] (*N *= 265)	
	Telemedicine (*n *= 1688)		In-person (*n *= 321)	Telemedicine (*n *= 156)	In-person (*n *= 152)	Telemedicine (*n *= 60)	In-person (*n *= 205)
Study design	Retrospective cohort			Retrospective cohort		Retrospective cohort	
Intervention	Phone follow-up			Phone follow-up		Phone or Video preoperative consultations	
Country	United States			United States		United States	
Study duration	2013-2019			2019-2021		2020-2023	
Follow-up duration	24 months			3 months		3 months	
Age (years)*	≤45: 454 (26.9%); 46-55: 336 (19.9%); 56-65: 470 (27.8%); >65: 428 (25.4%)	≤45: 74 (23.1%); 46-55: 66 (20.6%); 56-65: 100 (31.2%); >65: 81 (25.2%)		65 (Q1 52.5-Q3 73; 25-89)	67 (Q1 53.5-Q3 72.5; 21-88)	62 [49-71]	57 [44-69]
BMI kg/m^2^)*	≤25: 587 (34.9%); 25-30: 783 (46.6%); >30: 311 (18.5%)	≤25: 120 (37.85%); 25-30: 137 (43.22%); >30: 60 (18.93%)		25.8 (Q1 23.5-Q3 28.3; 16.4-39.4)	25.5 (Q1 23.0-Q3 29.0; 18.1-42.4)	25 [23-29];	25 [24-27]
MIS hernia surgery (laparoscopic or robotic) (%)	85%	53%		57%	39%	85%	80%
Open hernia surgery (%)	15%	47%		43%	61%	15%	20%

Pooled Analysis of All Studies

**Figure 2:**
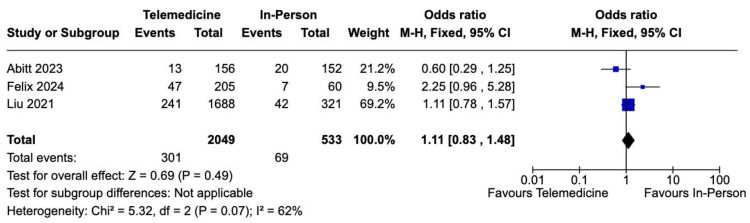
Overall complications analysis. Studies included: [5-7]. CI, confidence interval

Complication rate: All studies reported the incidence of different complications, such as SSI, SSO, urinary catheterization, pneumonia, urinary tract infection (UTI), sepsis, etc. Of the 2,582 patients, 370 (14%) experienced complications in both the telemedicine and in-person care groups. Between both groups, our analysis of complications did not reveal any statistically significant difference (14% vs. 13%; OR 1.11; 95% CI 0.83-1.48; *P *= 0.49; *I*² = 62%; Figure 2).

Surgical Site Occurrences

**Figure 3:**
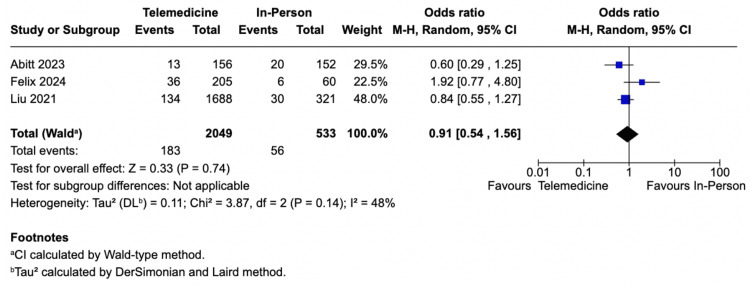
Surgical site occurrences analysis. Studies included: [5-7]. CI, confidence interval

All studies described different types of SSOs. Furthermore, they occurred in 258 patients (9.2%) and represented the most common complication. No significant difference was observed in the incidence of this outcome (SSOs) between the telemedicine and in-person interventions (8% vs. 10%; OR 0.91; 95% CI 0.54-1.56; *P *= 0.14; *I*² = 0.48%; Figure 3).

Hospital Readmissions

**Figure 4:**
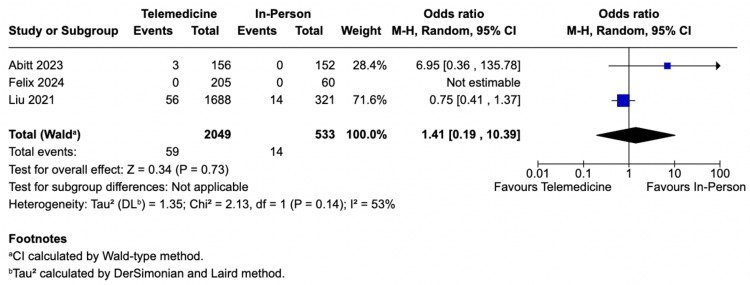
Hospital readmissions analysis. Studies included: [5-7]. CI, confidence interval

Similarly, the incidence of hospital readmissions did not differ significantly between the Telemedicine intervention and the control cohorts. The pooled analysis included only two studies [6,7] because Felix et al. [5] reported zero readmissions in both groups, making the OR not estimable. The pooled estimate from the two studies (OR 1.41; 95% CI 0.19-10.39; *P *= 0.73; *I*² = 53%; Figure 4) should be interpreted with caution, as it is essentially a weighted average of only two studies. 

Risk of Bias Assessment

The risk of bias for the included non-randomized studies was assessed independently by two authors (RC and AC) using the Risk Of Bias In Non-randomized Studies of Interventions (ROBINS-I) tool. This tool evaluates bias across seven domains: (1) bias due to confounding, (2) bias in selection of participants, (3) bias in classification of interventions, (4) bias due to deviations from intended interventions, (5) bias due to missing data, (6) bias in measurement of outcomes, and (7) bias in selection of the reported result.

Each domain was assessed as having low, moderate, serious, or critical risk of bias based on predefined criteria. Discrepancies in scoring were resolved through discussion and consensus. Overall, two of the included studies were judged to have a moderate risk of bias, primarily due to their retrospective design and potential unmeasured confounding factors, while one study was rated as having a low risk of bias. None of the studies were rated as having a critical risk of bias.

**Figure 5:**
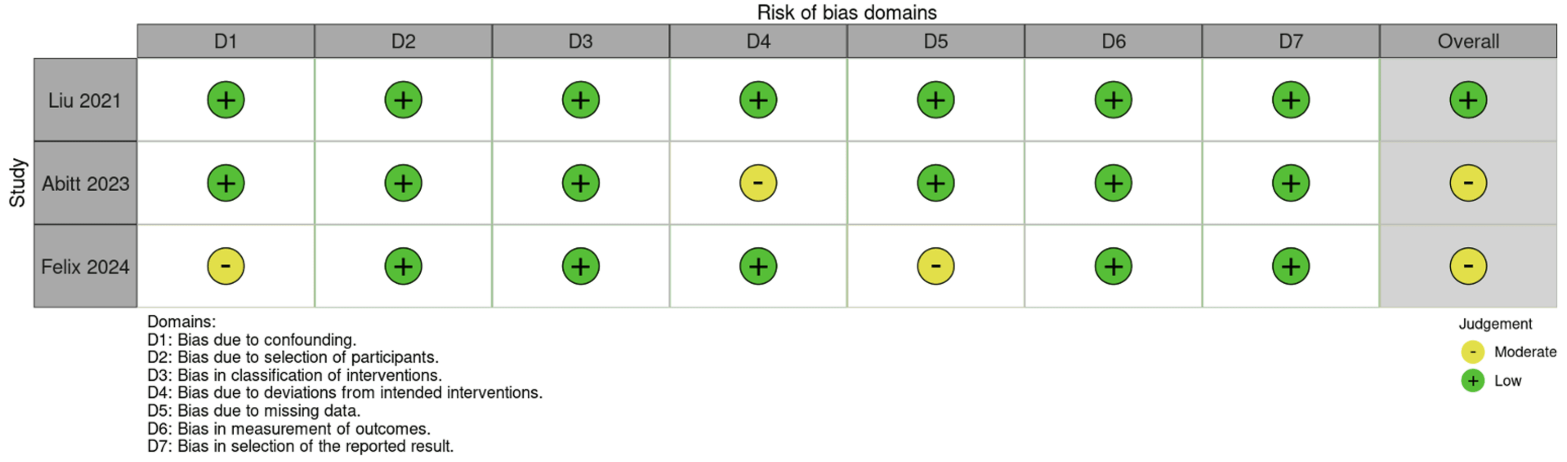
Risk of bias assessment for included studies according to the ROBINS-I tool across seven domains. Studies included: [5-7]. CI, confidence interval; ROBUNS-I, Risk Of Bias In Non-randomized Studies of Interventions

A detailed summary of the ROBINS-I assessments for each study is presented in Figure 5.

Discussion

In this systematic review and meta-analysis of three studies and 2,582 patients, we compared perioperative telemedical interventions to traditional in-person perioperative care for patients undergoing hernia surgery. The main findings were: (1) the same rate of complications between both groups, (2) no significant difference in the rate of SSOs, and (3) the same rate of readmissions in patients with perioperative telemedicine care.

Telemedicine has become more popular during the last few years across many medical specialties such as dermatology [13], radiology [14], cardiology [15,16], and endocrinology [17], with satisfactory results [18]. Its use expanded significantly during the global COVID-19 pandemic, serving as an alternative to reduce unnecessary hospital visits and minimize patient exposure [19].

However, due to the nature of surgical consultations, only a limited number of studies have evaluated the role of telemedicine in the surgical field [13,18,20], and surgical perioperative visits remain largely face-to-face, often contributing to overcrowded clinics. Updating perioperative protocols could help reduce costs while preserving outcomes and patient satisfaction [19,20]. There are more than 700,000 inguinal hernia and 400,000 ventral hernia repairs being performed in the United States every year [21]. Not only is hernia surgery one of the most frequent procedures performed worldwide, but it also has a relatively low incidence of complications, which is even more accentuated with the advancements in minimally invasive surgery [22].

Nowadays, there is an ongoing debate among surgeons regarding telemedicine interventions in hernia surgery. While various aspects of this approach have been explored, most studies remain exploratory and lack a comparator group. For instance, Eisenberg et al. described a telehealth follow-up intervention after laparoscopic inguinal hernia repair, showing that only 9% of patients requested an additional in-person evaluation, and 90% of the patients approved the digital care they received [23].

From another perspective, a frequently cited concern among surgeons opposed to telemedicine is the absence of physical examination. However, it is very well described that, specifically for hernias, phone questionnaires can be performed to identify complications such as recurrences with a sensitivity of 100% and specificity of 86% [24,25]. Considering these findings, telemedicine interventions could have a positive impact by reducing travel time for patients, costs for health provider institutions, and overcrowding in medical offices.

Liu et al. described that the use of a telephone follow-up two to three weeks after surgery was a safe and reasonable substitute for routine face-to-face clinic follow-up after analyzing the same rate of complications between both groups (14% vs. 13.5%) [6]. The same intervention was described by Abbitt et al. in 308 patients with similar results (8.3% vs. 13.1%) [7].

In abdominal wall surgery, wound complications, particularly SSOs, are among the most common postoperative issues [26-28]. In our analysis, Liu et al. [6] reported an overall SSO rate of 8.1%, with 7% in the telemedical follow-up group and 9% in the traditional in-person care group (*P* > 0.05). Abbitt et al. [7] and Felix et al. [5] reported SSO rates of 10.7% and 15.8%, respectively. Across all included studies, no statistically significant differences were observed between telemedical and conventional care.

Despite being the most frequently observed complication, SSOs may not have a significant impact on patient-centered outcomes. For instance, Miller et al. [29] examined 867 patients undergoing ventral hernia repair and found that postoperative pain and hernia-related quality of life at both 30 days and one year were comparable between patients who developed a seroma and those who did not. Furthermore, composite hernia recurrence rates were similar between groups (17% in the seroma group vs. 18% in the non-seroma group; *P* = 0.80).

Although complications such as seroma formation or wound issues can lead to hospital readmissions, overall readmission rates were low across all included studies. Liu et al. [6] and Abbitt et al. [7] reported 2.8% and 2.6% readmissions, respectively, with no notable differences between telemedicine and traditional in-person follow-up. Felix et al. reported zero readmissions in both groups, calculating an OR not possible [5]. Given that only two studies had estimable data for meta-analysis, and one study could not be included, these findings should be interpreted with caution. Overall, hospital readmissions appear uncommon in patients undergoing hernia surgery regardless of follow-up modality, but definitive conclusions cannot be drawn due to the limited number of studies and low event rates. Liu et al. [6] did not specify the causes of readmissions in their analysis, whereas Abbitt et al. [7] reported that readmissions were related to deep SSI and hematoma evacuation.

Despite these promising findings, our study includes limitations. First, the number and type of studies included limit the strength of our conclusions. All three studies were retrospective in nature and not randomized controlled trials, which may introduce bias and affect the level of evidence. Also, the included studies did not consistently report the type of hernia repair (inguinal vs. incisional vs. ventral). As a result, our findings should be interpreted as exploratory and applicable to abdominal wall hernia repairs broadly. Nevertheless, all included studies were published within the last five years, ensuring that the telemedical interventions analyzed reflect current clinical practice in institutions that have integrated this technology.

Second, there was variability, such as the telemedical interventions. While all studies involved a digital component, the interventions were not standardized. Two studies [6,7] specifically examined digital follow-up in the postoperative setting, whereas one study [5] focused on preoperative virtual consultations. This heterogeneity may influence the comparability of the results. However, the inclusion of both preoperative and postoperative interventions is justified, as all perioperative telehealth strategies aim to reduce patient burden, minimize costs, and improve surgical outcomes and engagement.

Third, due to the limited number of included studies (*n *= 3), we were unable to reliably assess publication bias using a funnel plot, and this represents a limitation of our analysis. Consequently, the possibility of undetected publication bias cannot be excluded. It should be noted that the large sample size of Liu et al. [6] heavily influenced the pooled results, with the smaller studies contributing relatively little. Finally, the included studies did not report standardized information on hernia type, size, procedure type, or postoperative drain use. These factors can affect postoperative outcomes and should be considered in future studies evaluating telemedicine follow-up in hernia surgery.

## Conclusions

Perioperative telemedicine in hernia surgery may be safer in comparison to traditional in-person care, without significant differences in complications, readmissions, or SSOs. Our findings support digital perioperative care as a potentially feasible, resource-efficient alternative that enhances patient access without increasing complications or compromising surgical outcomes. However, the feasibility of implementation and cost-effectiveness were not directly assessed in the included studies. Future randomized controlled trials are needed to further evaluate these aspects and long-term outcomes.
